# Real-World Outcomes of Primary Versus Interval Debulking Surgery in a Multicenter Cohort of Advanced Ovarian Cancer Patients Treated with Bevacizumab

**DOI:** 10.3390/cancers18050805

**Published:** 2026-03-02

**Authors:** Kaja Michalczyk, Lubomir Bodnar, Marta Czeluścińska-Murawiec, Anna Dańska-Bidzińska, Paweł Derlatka, Beata Maćkowiak-Matejczyk, Wioleta Sawczuk, Barbara Radecka, Edyta Operacz, Szymon Piątek, Ewa Kalinka, Adam Miller, Anita Chudecka-Głaz

**Affiliations:** 1 Department of Gynecological Surgery and Gynecological Oncology of Adults and Adolescents, Pomeranian Medical University, 70-204 Szczecin, Poland; anita.chudecka.glaz@pum.edu.pl; 2 Department of Clinical Oncology and Radiotherapy, St. John Paul 2nd Mazovia Regional Hospital in Siedlce, 08-110 Siedlce, Poland; lubomir.bodnar@uws.edu.pl (L.B.); mczeluscinska@szpital.siedlce.pl (M.C.-M.); 3 Faculty of Medical and Health Sciences, University of Siedlce, 08-110 Siedlce, Poland; 4 Department of Gynecologic Oncology, Maria Sklodowska-Curie National Research Institute of Oncology, 00-001 Warsaw, Poland; ad.bidzinska@gmail.com (A.D.-B.); pawel.derlatka@nio.gov.pl (P.D.); szymon.piatek@aol.com (S.P.); 5 Second Department of Obstetrics and Gynecology, Medical University of Warsaw, 02-091 Warsaw, Poland; 6 Department of Gynaecological Oncology, Maria Sklodowska-Curie Bialystok Oncology Centre, 15-027 Bialystok, Poland; bmackowiak@onkologia.nazwa.pl (B.M.-M.); wsawczuk@onkologia.bialystok.pl (W.S.); 7 Department of Oncology, Institute of Medical Sciences, University of Opole, 45-040 Opole, Poland; barbara.radecka@uni.opole.pl (B.R.); edyta.operacz@uni.opole.pl (E.O.); 8 Tadeusz Koszarowski Cancer Centre in Opole, 45-061 Opole, Poland; 9 Department of Oncology, Polish Mother’s Memorial Hospital Research Institute, 93-338 Lodz, Poland; ewa.kalinka@iczmp.edu.pl (E.K.); adam.miller@iczmp.edu.pl (A.M.)

**Keywords:** PDS, IDS, NACT, ovarian cancer, bevacizumab

## Abstract

Background: With an ongoing debate concerning the optimal timing of advanced ovarian cancer surgical treatment, primary debulking surgery (PDS) versus neoadjuvant chemotherapy followed by interval debulking surgery (IDS), this study aimed to compare survival outcomes between PDS and IDS populations and evaluate prognostic factors in a real-world cohort of patients treated with first-line chemotherapy and bevacizumab. Methods: A retrospective multi-center study was conducted involving 369 patients with newly diagnosed advanced ovarian cancer. Patient data included demographics, histology, treatment details, chemotherapy response, and survival outcomes. Kaplan–Meier estimates with log-rank tests were used for univariate analyses, as well as Cox proportional hazard models for multivariate analyses. Results: Patients undergoing IDS were older (62.5 vs. 60.0 years), had higher pretreatment CA-125 (1846 vs. 395.6 IU/mL), an increased proportion were at with stage IV (36.25% vs. 21.10%), and they received fewer bevacizumab cycles (12 vs. 18) compared to those undergoing PDS. Median progression-free survival (PFS) was 18.6 months (95% CI: 17.3–20.2) and overall survival (OS) was 45.4 months (95% CI: 41.1–52.1). Multivariate analysis confirmed poor chemotherapy response (HR 1.80, 95% CI: 1.36–2.37; *p* < 0.0001) and IDS (HR 1.65, 95% CI: 1.37–2.39; *p* < 0.0001) as independent predictors of shorter PFS. For OS, independent risk factors were age > 70 (HR 1.62; *p* = 0.0202), poor response (HR 2.03; *p* < 0.0001), and IDS (HR 1.75; *p* = 0.0006). Conclusions: In this real-world cohort treated with first-line chemotherapy and bevacizumab, interval debulking surgery was associated with inferior progression-free and overall survival compared with primary debulking surgery. However, these findings reflect a high-risk population and are strongly influenced by patient selection and treatment pathways, underscoring the need for cautious interpretation.

## Simple Summary

This study investigated the real-world outcomes of two surgical approaches for advanced ovarian cancer—primary debulking surgery (PDS) and interval debulking surgery (IDS)—in patients receiving first-line chemotherapy with bevacizumab. Patients who underwent IDS were typically older, had higher initial CA-125 levels, and presented with more advanced (stage IV) disease compared to those receiving PDS. The key finding was that IDS was independently associated with significantly poorer progression-free survival (PFS) and overall survival (OS) compared to PDS. Poor chemotherapy response and age over 70 years were also identified as independent predictors of worse outcomes.

## 1. Introduction

Advanced ovarian cancer (AOC) remains a significant challenge in gynecologic oncology. Despite an aggressive multimodal therapeutic approach, patient prognosis is still limited, with the 5-year overall survival rates for metastatic disease at around 30% [1]. The standard of care for newly diagnosed epithelial ovarian cancer combines maximal surgical cytoreduction with systemic, platinum-based chemotherapy. The volume of residual disease following surgery is one of the most powerful independent prognostic factors in AOC [2,3]. For patients whose disease burden is deemed too extensive for optimal upfront cytoreduction, or for those with a high perioperative risk profile, neoadjuvant chemotherapy (NACT) may be administered to shrink tumors, followed by interval debulking surgery (IDS) [4,5]. A central, ongoing debate concerns the optimal timing of surgery: primary debulking surgery (PDS) performed before chemotherapy versus neoadjuvant chemotherapy followed by IDS. While landmark trials like EORTC 55971 and CHORUS established IDS as a non-inferior alternative to PDS, particularly for patients unlikely to achieve optimal cytoreduction upfront, this conclusion has been challenged by recent evidence suggesting a potential benefit for PDS.

The therapeutic landscape has further evolved with the integration of targeted agents. The addition of bevacizumab, an anti-angiogenic agent, to first-line chemotherapy became a standard of care following the pivotal GOG-0218 [6] and ICON7 [7] trials. Both trials successfully met their primary endpoint, demonstrating a statistically significant, albeit modest, improvement in progression-free survival (PFS) for the arms that included maintenance bevacizumab. Despite the PFS benefit, the secondary endpoint of overall survival (OS) of the final analyses did not show any benefit of the intent-to-treat populations of either trial. In ICON7, an exploratory analysis was conducted on a “high-risk” group, defined as those with stage IV disease, suboptimally debulked stage III disease with more than 1 cm of residual tumor [8]. In this population, the addition of bevacizumab demonstrated a significant OS benefit, with a median OS of 39.3 months versus 34.5 months for chemotherapy alone. The PFS benefit was also more pronounced in this group, with a median of 15.9 months versus 10.5 months. Although not prospectively defined, the final OS analysis of GOG-0218 revealed a similar finding for high-risk patients. Among patients with stage IV disease, the Bev-Maintenance arm was associated with a median OS of 42.8 months, a 10-month improvement over the 32.6 months seen in the control arm (HR 0.75; 95% CI, 0.59–0.95) [9]. The trial results indicating the benefit of concurrent and maintenance therapy in high-risk, advanced-stage disease led to regulatory approvals and clinical practice guidelines worldwide, including the creation of the Polish reimbursement program allowing for first-line and maintenance bevacizumab treatment [10]. However, the interplay between the choice of surgical approach (PDS vs. IDS) and the use of bevacizumab in a real-world setting is still not fully understood.

This study presents a retrospective analysis of patients with advanced-stage (FIGO III–IV) ovarian cancer, all of whom were treated with first-line chemotherapy and bevacizumab. The primary objective is to compare survival outcomes (PFS and OS) between patients undergoing PDS versus IDS in a contemporary, real-world cohort, with a secondary aim to evaluate prognostic factors associated with survival.

## 2. Materials and Methods

This was a retrospective real-world evidence analysis of the treatment of patients with advanced ovarian cancer as part of the Polish government’s drug program B.50 announced by the Ministry of Health on the list of reimbursed medicines. As a part of the treatment program, patients received a regimen of standard paclitaxel 175 mg/m^2^ with carboplatin AUC 5–6 every 21 days (CP) with bevacizumab 7.5 mg/kg given concurrently on day 1 every 3 weeks, followed by maintenance bevacizumab for a total of up to 18 cycles or until the progression of the disease. The drug program specified patient eligibility criteria for the treatment, criteria for exclusion from the program, drug dosage regimen, method of drug administration, a list of diagnostic tests performed when qualifying a patient for the program, and procedures necessary for monitoring the treatment. The specific program criteria are attached in Appendix A.

Data were gathered from seven major Polish gynecologic oncology treatment centers. This study was supported by PTGO (Polish Society of Gynecologic Oncology) and included patients with a histological diagnosis of advanced ovarian cancer, fallopian tube cancer, or primary peritoneal cancer; FIGO stages IV or III (with residual disease after cytoreduction ˃ 1 cm (suboptimal cytoreduction)); and an elevated pretreatment CA-125 serum level. Notably, patients undergoing IDS who achieved optimal cytoreduction (≤1 cm) were excluded from this analysis based on the program inclusion criteria, resulting in a high-risk IDS cohort with suboptimal debulking. All patients included in the study underwent either primary cytoreductive or interval debulking surgery. Patients included in the registry could not have had any previous systemic treatment for ovarian cancer, with the exception of neoadjuvant chemotherapy (NACT), which was a mandatory inclusion criterion for the IDS arm. NACT typically consisted of three to four cycles of paclitaxel and carboplatin prior to surgery. Patients with unavailable or incomplete clinicopathological data were excluded from the study. Patients’ age, tumor histology, type of chemotherapy (neoadjuvant/adjuvant), number of cycles, surgery date, surgical outcomes (optimal/suboptimal cytoreduction/no surgery), and chemotherapy response (RECIST 1.1. criteria [11]) were collected. Sample size was determined by the available retrospective registry data within the study period. While no a priori power calculation was performed, the inclusion of 369 patients was considered sufficient to provide descriptive estimates of survival differences, though the imbalance between PDS (n = 289) and IDS (n = 80) groups may limit the statistical precision of comparative analyses.

### Statistical Analysis

PFS was defined as the time from ovarian cancer diagnosis (either during diagnostic laparoscopy/biopsy or cytoreductive surgery) to disease progression or last follow-up; OS was the time from ovarian cancer diagnosis to death or last follow-up. The cut-off date was December 2024. Group comparisons were performed using the Mann–Whitney U or χ^2^ test, as appropriate. Survival was estimated by the Kaplan–Meier method and compared with the log-rank test. A two-sided *p* < 0.05 was considered significant. Multivariate analyses of PFS and OS were performed using Cox proportional hazard regression (forward stepwise). The proportional hazard assumption was verified using Schoenfeld residuals. All prespecified covariates (surgical outcomes, age, FIGO stage, histopathology, and response to chemotherapy) were included in the univariate and multivariate models. CA-125 was analyzed descriptively but excluded from survival models due to missing data points in >10% of the cohort. The proportional hazard assumption for all covariates included in the final models was assessed graphically using log–log plots and statistically using Schoenfeld residuals. No significant violations of the proportional hazard assumption were detected for any included variable. All of the patients were included in survival analyses. Analyses were conducted using MedCalc software (v23.3.7; Ostend, Belgium).

## 3. Results

### 3.1. Patient Characteristics

This was a database created based on the Polish National B50 program that had specific inclusion criteria (listed in Appendix A). Among seven gynecologic centers participating in the study, 388 patients were registered in the database. Nineteen patients were not included in the final cohort (n = 369) due to the absence of cytoreductive surgery. The final cohort included 289 patients undergoing primary debulking surgery and 80 receiving interval debulking surgery. The median age was 60 years (95% CI: 42–75), with 88% of patients aged ≤ 70 years. The majority had a serous histological subtype (92%). Most patients were diagnosed at FIGO stage III (76%), while 24% were at stage IV. PDS was performed in 78% of cases, while IDS was performed in 22% of patients. Responses to chemotherapy were the following: complete response (CR) in 27%, partial response (PR) in 49%, stable disease (SD) in 15%, and progressive disease (PD) in 6%. The median CA-125 level prior to treatment was 494 U/mL (95% CI: 66–6609), and the median number of bevacizumab cycles administered was 18 (95% CI: 4–18). Patient distribution per center is detailed in Supplementary Table S1. No single center contributed >30% of the population, minimizing center-specific bias.

### 3.2. Comparison of Characteristics by Surgery Type

Significant differences were observed between patients undergoing PDS and IDS. The median age was slightly lower in the PDS group (60 years, 95% CI: 42–74) compared with IDS (62.5 years, 95% CI: 41–77.5; *p* = 0.0099). Pretreatment CA-125 levels were markedly higher in the IDS group (median 1846 U/mL, 95% CI: 101–12,140) compared with PDS (395.6 U/mL, 95% CI: 47–4901; *p* < 0.0001). FIGO stage IV was more common in IDS (36%; 29/80 patients) compared with PDS (21%; 61/289 patients; *p* = 0.0053). Serous histology predominated in both groups, although non-serous subtypes were less frequent in IDS (*p* = 0.0207). The median number of bevacizumab cycles was higher in PDS (18, 95% CI: 5–18) compared with IDS (12, 95% CI: 3–18; *p* < 0.0001). Chemotherapy responses (PR/CR vs. PD/SD) were more favorable in PDS n = 278; 80% PR/CR [n = 222] vs. 20% PD/SD [n = 56] than in IDS (n = 77; 74% PR/CR [n = 57] vs. 26% PD/SD [n = 20]; *p* < 0.0001). These comparisons are summarized in Table 1.

### 3.3. Factors Associated with Progression-Free and Overall Survival

In the univariate analysis, those with a poor chemotherapy response (PD/SD vs. PR/CR) and undergoing IDS were significantly associated with shorter PFS (*p* < 0.0001 for both). Age, FIGO stage, and histology were not significant predictors. The multivariate analysis confirmed that poor chemotherapy response (HR 1.80; *p* < 0.0001) and IDS (HR 1.65; *p* < 0.0001) were independent predictors of worse PFS, while age, FIGO stage, and histological subtype remained non-significant (Table 2).

As for overall survival, the univariate analysis identified several factors associated with shorter OS: age > 70 years (*p* = 0.0244), poor chemotherapy response (*p* < 0.0001), IDS (*p* < 0.0001), and FIGO stage IV (*p* = 0.0380). The histological subtype was not associated with OS (*p* = 0.9171).

In the final multivariate model, three factors remained independent predictors of worse OS: age > 70 (HR 1.62; *p* = 0.0202), poor chemotherapy response (HR 2.03; *p* < 0.0001), and IDS (HR 1.75; *p* = 0.0006). FIGO stage and histological subtype were not independently associated with OS (Table 2).

A post hoc sample size calculation was performed to determine the significance of the results. Based on median patient survival (PDS of 52.0 months vs. IDS of 27.5 months) and the hazard ratio of 1.75, the study power had over >99% power to detect the difference in median survival at a significance level of 0.05.

### 3.4. Patient Survival Outcomes and the Impact of Surgical Timing on Survival

**Figure 1:**
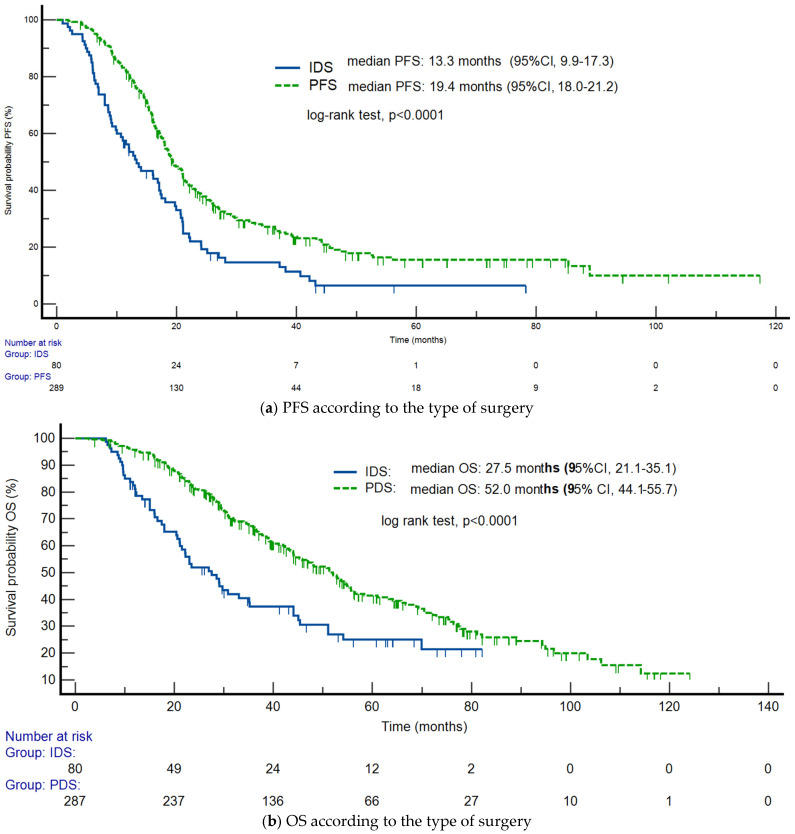
Kaplan–Meier PFS and OS curves. (**a**) PFS according to the type of surgery (PDS vs. IDS). (**b**) OS according to the type of surgery (PDS vs. IDS).

For the entire study cohort, the median progression-free survival (PFS) was 18.6 months (95% CI: 17.3–20.2), and the median overall survival (OS) was 45.4 months (95% CI: 41.1–52.1). The timing of surgery had a profound impact on survival outcomes, as illustrated in the following figures. Figure 1a illustrates the estimated progression-free survival using Kaplan–Meier curves for the IDS and PDS groups. Patients who underwent PDS had a significantly longer median PFS of 19.4 months compared to 13.3 months for those who had IDS (log-rank test, *p* < 0.0001). Figure 1b visualizes the substantial difference in patients’ OS based on surgery type. The median OS for the PDS group was nearly double that of the IDS group, with a highly significant difference of 52.0 months versus 27.5 months (log-rank test, *p* < 0.0001).

## 4. Discussion

The results of this retrospective analysis of advanced ovarian cancer patients treated with first-line chemotherapy and bevacizumab provide valuable real-world insights into the first-line treatment of ovarian cancer.

A key finding of this study is the significantly inferior progression-free survival and overall survival in patients undergoing interval debulking surgery compared to primary debulking surgery. The results of this study can be contextualized by the recent findings of the landmark TRUST trial [12,13] as they are consistent with its secondary endpoint, which, for the first time in a large, randomized controlled setting, demonstrated a statistically significant PFS benefit for PDS over IDS. In our study, the median PFS of 18.6 months and OS of 45.4 months for the entire cohort in this analysis are noteworthy and similar to the results of the TRUST trial (19.7 months for IDS and 22.2 months for PDS). However, the overall survival of patients noted in our study is lower than reported in the TRUST trial (48.3 months for IDS and 54.3 months for PDS), which could be due to the inclusion of patients who may not have met the strict eligibility criteria of the TRUST trial, such as those with poorer performance status or more extensive disease.

While the primary endpoint of OS in the TRUST trial did not show a statistically significant difference, the trend favored the PDS approach. The more pronounced survival difference in this retrospective cohort may be attributable to selection bias, as patients triaged to IDS in a real-world setting often present with a higher tumor burden and more comorbidities, making them poorer candidates for upfront surgery. This was reflected in the baseline characteristics of the IDS group in this study, which had a higher proportion of FIGO stage IV disease and significantly higher pretreatment CA-125 levels. This real-world context is further illuminated by Table S1 in the Supplementary Materials, which details the distribution of patients across all of the study centers. The notable variation in both the number of patients contributed by each center and the widely differing proportions of IDS versus PDS patients within these centers (ranging from 0% IDS in one center to 33% in another) visually reinforces the presence of this real-world selection bias. This suggests that patient allocation to either PDS or IDS was likely influenced by center-specific factors, such as local clinical judgment, institutional capabilities, or the characteristics of patients presenting at each site, thereby contributing to the observed differences in baseline patient features between the two surgical groups. The observed inferior outcomes in the IDS group in this retrospective analysis must be interpreted with caution. A major limitation is the exclusion of optimal responders in the IDS group due to registry criteria. By design, this study excludes IDS patients who responded well to neoadjuvant chemotherapy and achieved optimal cytorectuction (≤1 cm) during interval debulking surgery, while the PDS arm includes patients who might have been platinum-sensitive as well as platinum-resistant. This systematically biases the IDS cohort toward chemotherapy-resistant or poor responders—a group already known to have intrinsically poor prognosis. Consequently, the observed survival disadvantage of IDS largely reflects tumor biology and preselection rather than surgical timing itself. These findings apply specifically to a high-risk population and likely overestimate the survival deficit associated with IDS compared to randomized trials that include optimal responders.

The multivariate analysis identified poor chemotherapy response by RECIST 1.1 criteria [11] as a strong prognostic factor for both PFS and OS, alongside age > 70 and the choice of IDS. While the statistical independence of RECIST 1.1. response within the Cox model suggests its strong predictive value after accounting for other factors, it is crucial to interpret this finding with nuance. Chemotherapy response, by its nature, is not a static baseline characteristic but rather an intermediate outcome reflecting the underlying disease biology and the effectiveness of the initial neoadjuvant treatment. Given that patients triaged to IDS often presented with a higher tumor burden and more advanced disease (as reflected by higher CA-125 levels and increased stage IV cases in our cohort), a “poor chemotherapy response” in this group may, to some extent, be a manifestation of inherently more aggressive or resistant disease that also influenced the initial decision for IDS. This complex interplay underscores that while RECIST 1.1. response serves as a robust predictor, its significance is deeply linked to both the initial disease state and the efficacy of the treatment strategy. This highlights the ongoing challenge of patient selection and the need for more refined predictive markers to identify patients who will truly benefit most from neoadjuvant chemotherapy versus upfront surgery.

Several trials were conducted to compare primary vs. interval debulking surgery approaches. The results of EORTC 55971 [14] and CHORUS trials [15,16] established neoadjuvant chemotherapy followed by IDS as a viable alternative to PDS. The results of the EORTC 55971 trial showed that neoadjuvant chemotherapy followed by IDS was not inferior to PDS in terms of OS and PFS for patients with bulky stage IIIC or IV ovarian cancer. The CHORUS trial came to a similar conclusion as the EORTC study, finding that OS and PFS were comparable between the PDS and IDS arms. It also noted lower rates of surgical morbidity and mortality in the IDS group. However, their findings have been debated, particularly regarding surgical quality control. A key factor that distinguishes our study from the previous clinical trials is that the entire patient cohort received bevacizumab, which was not a standard component of therapy in the EORTC, CHORUS, or TRUST trials. The interaction between bevacizumab and the timing of surgery could contribute to the different outcomes. Moreover, in our study, the IDS group had more adverse prognostic factors at baseline (older age, higher CA-125, more stage IV disease). Another critical consideration is the perioperative morbidity profile. Lower perioperative morbidity is a well-established benefit of IDS and a key determinant in clinical decision-making, particularly for patients with high tumor burden or comorbidities. A significant limitation of this study is the absence of detailed data on perioperative complications and morbidity rates for either surgical approach. Without this data, we cannot fully assess the trade-offs between the potential survival benefits of PDS and the reduced surgical risks typically associated with IDS. Consequently, the comparison presented here focuses strictly on survival outcomes and does not capture the full clinical picture regarding surgical safety. This reflects a “real-world” study selection bias where patients with a greater disease burden or with multiple comorbidities often are triaged to the IDS group. A recent meta-analysis of five phase III randomized controlled trials in FIGO III and IV advanced ovarian cancer patients showed no difference in survival outcomes between NACT-IDS and PDS patients, while patients undergoing IDS had higher complete resection rates and reduced surgical morbidity [17]. What needs to be underlined is that in our study, only patients who had tumor residues after debulking surgery (either PDS or IDS) were included in the study and survival analysis. In accordance with ESMO guidelines, patients with advanced ovarian cancer should be evaluated for primary cytoreductive surgery with the aim of achieving complete cytoreduction, and if complete cytoreduction is feasible, PCS is recommended. Otherwise, interval treatment should be considered [5].

This real-world evidence study provides valuable insights into the effectiveness of first-line bevacizumab in the high-risk ovarian cancer population. The findings align with and further support previously established data regarding the role of bevacizumab treatment in this setting [18,19]. In accordance with ESMO guidelines, the use of bevacizumab in stages III–IV ovarian cancer patients should be considered as it improves PFS [5,20]. It is also one of the preferred regimens suggested by NCCN for the primary systematic therapy for stages II–IV patients [21]. The results of our study demonstrate similar progression-free survival. The median PFS of high-risk patients defined as FIGO stage IV disease or suboptimally debulked stage III disease with more than 1 cm of residual tumor receiving first-line CP chemotherapy with concurrent and maintenance bevacizumab treatment was 18.6 months, which is consistent with the results from the bevacizumab arms of both GOG-0218 [6] (which reported a median PFS of 18.2 months for the bevacizumab-throughout arm) and ICON7 [7], suggesting a comparable response to the chemotherapy–bevacizumab combination to those in the pivotal registration trials.

The survival outcomes in our study align with the bevacizumab-treated population based on GOG-0218 and ICON7. However, our findings on the superiority of PDS over IDS challenge the older conclusions from EORTC and CHORUS trials. The results of our study align more closely with the recent evidence from the TRUST trial, suggesting that in the modern era of targeted therapy and improved surgery, upfront cytoreduction may offer a greater benefit.

## 5. Conclusions

In conclusion, this study provides valuable real-world evidence that complements the findings of the TRUST trial. It confirms the prognostic importance of surgical timing, response to chemotherapy, and age in advanced ovarian cancer. The observed inferior outcomes in the IDS group in this retrospective analysis must be interpreted with caution. The lack of perioperative morbidity data limits our ability to evaluate the relative safety profiles of the two approaches. Due to the exclusion of optimal responders in the IDS arm, the findings apply specifically to a high-risk, poor-responder IDS population. Furthermore, this study does not provide novel prognostic insight regarding surgical timing alone, as the survival disadvantage likely stems from the fact that poor response to chemotherapy is already well established as a negative prognostic factor. Consequently, these findings may overestimate the survival deficit associated with IDS compared to trials including optimal responders. The results do, however, serve as real-world evidence corroborating the PFS benefit established by landmark trials for adding bevacizumab to first-line therapy. Future research should continue to focus on refining the criteria for selecting patients for each approach and on developing novel therapeutic strategies to improve outcomes for all patients with advanced ovarian cancer.

## Figures and Tables

**Table 1:** Comparison of patient characteristics and outcomes by type of surgery (PDS vs. IDS) (n = 369).

PARAMETER	PDS	IDS	*p* Value
	N = 289	N = 80	
Age, years median (95% CI)	60 (42–74)	62.5 (41–77.5)	0.0099 ^a^
CA 125 before treatment, median, (95% CI) U/mL	395.6 (47.0–4901.0)	1846.0 (101.0–12,140.0)	<0.0001 ^a^
FIGO stage: III IV	228 61	51 29	0.0053 ^b^
Histology: serous other	260 29	79 1	0.0207 ^c^
Median number of administered bevacizumab cycles (95% CI)	18 (5–18)	12 (3–18)	<0.0001 ^a^
RECIST 1.1. PD, SD PR, CR	56 222	20 57	<0.0001 ^b^

Abbreviations: ^a^—Mann–Whitney test; ^b^—Chi-square; ^c^—Chi-square; Yates corrected test; CA125—Cancer Antigen 125; RECIST—Response Evaluation Criteria in Solid Tumors; CR—Complete Response; PR—Partial Response; SD—Stable Disease; PD—Progressive Disease; IDS—Interval debulking surgery; PDS—Primary debulking surgery; FIGO—International Federation of Gynecology and Obstetrics. Although initial CA-125 levels were analyzed descriptively and showed significant differences between surgical groups, this variable was not included in the final multivariate Cox models. This decision was based on its high correlation with the FIGO stage (which was included and represents a broader measure of disease burden) and the potential for collinearity, which could obscure the independent effects of other key prognostic factors.

**Table 2:** Univariate and multivariate analyses of progression-free and overall survival.

Covariate	n (%)	PFS—Univariate Analysis		PFS—Multivariate Analysis		OS—Univariate Analysis		OS—Multivariate Analysis	
		Median (Months), 95% CI	*p* Value	HR (95% CI)	*p* Value	Median (Months), 95% CI	*p* Value	HR (95% CI)	*p* Value
**Age**									
≤70 years	324 (88%)	18.7 (17.4–20.7)	0.4430		0.5312	47.9 (43.1–54.1)	0.0244		0.0202
>70 years	45 (12%)	16.9 (15.7–20.9)		1.12 (0.79–1.60)		30.9 (22.2–51.3)		1.62 (1.07–2.44)	
**RECIST 1.1 response**									
PR/CR	279 (79%)	20.3 (18.1–21.4)	<0.0001		<0.0001	51.4 (44.1–55.7)	<0.0001		<0.0001
PD/SD	76 (21%)	13.3 (9.7–16.5)		1.80 (1.36–2.37)		27.9 (19.6–37.1)		2.03 (1.49–2.75)	
**Type of surgery**									
PDS	289 (78%)	19.4 (18.0–21.2)	<0.0001		<0.0001	52.0 (44.1–55.7)	<0.0001		0.0006
IDS	80 (22%)	13.3 (9.9–17.3)		1.65 (1.37–2.39)		27.5 (21.1–35.1)		1.75 (1.27–2.42)	
**FIGO stage**									
III	279 (76%)	19.1 (17.4–20.9)	0.1022		0.1935	47.1 (42.4–53.1)	0.0380		0.1204
IV	90 (24%)	17.1 (13.1–21.1)		1.20 (0.91–1.57)		35.9 (29.4–106.2)		1.28 (0.94–1.74)	
**Histology**									
Serous	339 (92%)	18.5 (17.1–19.8)	0.1825		0.1064	45.4 (41.1–52.0)	0.9171		0.9715
Non-serous	30 (8%)	21.9 (8.5–52.8)		1.47 (0.92–2.34)		52.1 (21.3–96.6)		0.99 (0.58–1.68)	

Abbreviations: CA125—Cancer Antigen 125; RECIST—Response Evaluation Criteria in Solid Tumors; CR—Complete Response; PR—Partial Response; SD—Stable Disease; PD—Progressive Disease; IDS—Interval debulking surgery; PDS—Primary debulking surgery; FIGO—International Federation of Gynecology and Obstetrics.

## Data Availability

The original contributions presented in this study are included in the article/Supplementary Materials. Further inquiries can be directed to the corresponding author.

## Supplementary Materials

The following supporting information can be downloaded at: https://www.mdpi.com/article/10.3390/cancers18050805/s1, Table S1. Distribution of patients among study centres.

## Appendix A

**Table A1:** B.50 Drug program—treatment of patients with advanced ovarian cancer using bevacizumab.

1.1. Qualification criteria	(1) Histological diagnosis of ovarian cancer, fallopian tube cancer, or primary peritoneal cancer; (2) FIGO stages IV or III with residual disease after cytoreduction ˃ 1 cm (suboptimal cytoreduction; description of residual neoplastic lesions left after surgery with size in centimeters is required); (3) No previous systemic treatment of ovarian cancer. Previous neoadjuvant chemotherapy is permissible; (4) General performance status stages 0–1 according to the Zubrod–WHO classification; (5) Age over 18 years; (6) Results of a blood count with a smear: (a) Platelet count greater than or equal to 1.5 × 105/mm^3^; (b) Absolute neutrophil count greater than or equal to 1500/mm^3^; (c) Hemoglobin concentration greater than or equal to 10.0 g/dL. (7) Coagulation parameters: (a) Activated partial thromboplastin time (APTT) within the normal range; (b) Prothrombin time (PT) or international normalized ratio (INR) within the normal range. (8) Liver and kidney function parameters: (a) Total bilirubin concentration not exceeding 2 times the upper limit of the norm (except for patients with Gilbert’s syndrome); (b) Transaminase activity (alanine and aspartate) in serum not exceeding 5 times the upper limit of the norm; (c) Creatinine concentration within the normal range. (9) Exclusion of pregnancy; (10) No contraindications to chemotherapy with carboplatin and paclitaxel; (11) No contraindications to the use of bevacizumab, which are: (a) Surgery performed within less than 4 weeks of qualification for treatment; (b) Active gastric or duodenal ulcer; (c) Unstable arterial hypertension; (d) Unstable ischemic heart disease; (e) History of vascular diseases of the central nervous system; (f) Congenital bleeding diathesis or acquired coagulopathy; (g) Medical conditions associated with an increased risk of bleeding; (h) Use of anticoagulants or antiplatelet drugs (excluding use in prophylactic doses); (i) Non-healing wounds; (j) Proteinuria; (k) Hypersensitivity to the drug or any of the excipients. The qualification criteria must be met cumulatively.
1.2. Determination of the duration of treatment in the program	Treatment lasts until the attending physician decides to exclude the beneficiary from the program, in accordance with the exclusion criteria.
1.3. Criteria for exclusion from the program	(1) Symptoms of hypersensitivity to bevacizumab; (2) Administration of 18 cycles of bevacizumab treatment; (3) Progression of the disease during treatment; (4) Long-term adverse effects of a degree equal to or greater than 3 according to the WHO classification; Persistent deterioration of general fitness. 1. Carboplatin with paclitaxel: (1) Carboplatin (AUC 5–6)—day 1; (2) Paclitaxel 175 mg/m^2^—day 1. Rhythm: every three weeks. Six cycles. 2. Bevacizumab −7.5 mg/kg body weight intravenously in a 30–90 min infusion—day 1. Rhythm: every 3 weeks. Eighteen cycles. (1) Patients will receive bevacizumab in combination with 3-week cycles of chemotherapy (maximum six cycles); (2) After completing chemotherapy, treatment will be continued in 3-week cycles until 18 cycles of bevacizumab treatment have been completed or until disease progression or unacceptable side effects occur (whichever comes first); (3) If chemotherapy or one of its components must be completed before completing six cycles of treatment, bevacizumab may be continued according to the principles described in point 2; (4) Bevacizumab will be administered from the first cycle of chemotherapy or from the second cycle if chemotherapy is started within 28 days of major surgery; (5) If it is necessary to discontinue carboplatin treatment, this drug can be replaced with cisplatin and treatment can be continued; (6) If a secondary surgical procedure is necessary, the procedure can be performed no earlier than 28 days after the administration of bevacizumab and the resumption of bevacizumab treatment cannot start earlier than 28 days after the surgical procedure; (7) Modifications of the dosage and rhythm of drug administration in accordance with the provisions of the relevant Summary of Product Characteristics. 1. Tests for qualification for bevacizumab treatment: (1) Histological confirmation of ovarian cancer, fallopian tube cancer, or primary peritoneal cancer; (2) Blood count with differential; (3) Serum concentration of: (a) Urea; (b) Creatinine; (c) Bilirubin. (4) Transaminase activity (AST, ALT); (5) Activated partial thromboplastin time (APTT); (6) INR or prothrombin time (PT); (7) CA125 concentration; (8) General urine test; (9) Pregnancy test—in women of childbearing potential; (10) Computed tomography of the abdomen and pelvis and other body areas depending on clinical indications; (11) Computed tomography or magnetic resonance imaging of the brain in case of clinical indications for imaging of metastases to the CNS; (12) Chest X-ray—if computed tomography of this area is not performed; (13) Electrocardiogram (ECG); (14) Blood pressure measurement; (15) Other tests if clinically indicated. Postoperative (before starting bevacizumab treatment) computed tomography of the abdomen and pelvis should be performed no earlier than 4 weeks after surgery, but no later than 2 weeks after starting chemotherapy. The purpose of initial imaging tests is to enable later monitoring of disease progression.
2. Monitoring the safety of bevacizumab treatment	(1) Blood count with differential; (2) Determination of the concentration of: (a) Creatinine; (b) Bilirubin—in serum; (c) APTT and PT or INR. (3) Determination of transaminase activity (AST, ALT); (4) General urine test; (5) Blood pressure measurement; (6) Other tests if clinically indicated. Tests are performed every 3 weeks or before starting the next cycle of therapy if the drug administration was delayed.
3. Monitoring the effectiveness of bevacizumab treatment	(1) Computed tomography of appropriate body areas depending on clinical indications; (2) Determination of CA125 concentration; (3) Other tests if clinically indicated. Computed tomography tests are performed: (1) After completion of chemotherapy; (2) During bevacizumab treatment: no less frequently than every 24 weeks; (3) At the time of exclusion from the program, if it occurred for reasons other than documented disease progression; (4) Always when the CA125 concentration increases above twice the nadir value; (5) Always when clinically indicated. CA125 concentration tests are performed no less frequently than every three treatment cycles: The assessment of treatment effectiveness is performed according to RECIST criteria.
4. Monitoring the implementation of the program	The president of the National Health Fund maintains a register of patients treated under the drug program, available via an online application.
