# Supplementary material for: Real-World Outcomes of Primary Versus Interval Debulking Surgery in a Multicenter Cohort of Advanced Ovarian Cancer Patients Treated with Bevacizumab
Source: Cancers (Basel). 2026 Mar 2;18(5):805. doi: 10.3390/cancers18050805 (PMC12984216; doi:10.3390/cancers18050805)
Supplement: Supplementary file 1 [file cancers-18-00805-s001.zip › cancers-4133953-supplementary.pdf]

Table S1. Distribution of patients among study centres

Study centre	N of patients	N of IDS patients	N of PDS patients
**Maria Sklodowska-Curie National Research Institute of Oncology, Warsaw, Poland**	44	12 (27%)	33 (73%)
**Pomeranian Medical University, Szczecin, Poland**	42	9 (21%)	33 (79%)
**Polish Mother's Memorial Hospital Research Institute, Lodz, Poland**	40	1 (2.5%)	39 (97.5%)
Tadeusz Koszarowski Cancer Centre in Opole, Poland	50	4 (8%)	46 (92%)
**St. John Paul 2nd Mazovia Regional Hospital in Siedlce, Poland**	12	0 (0%)	12 (100%)
**Maria Sklodowska-Curie Bialystok Oncology Centre, Bialystok, Poland**	72	18 (25%)	54 (75%)
**Second Department of Obstetrics and Gynecology, Medical University of Warsaw, Poland**	109	36 (33%)	73 (67%)
